# Hyaline Vascular Type of Castleman Disease: Diagnostic Pitfalls on Cytology and Its Clinical Relevance

**DOI:** 10.7759/cureus.17174

**Published:** 2021-08-14

**Authors:** Neha Singh, Nilotpal Chowdhury, Shweta Pal, Jagdish P Goyal, Bhanu Kiran Bhakhri, Shalinee Rao

**Affiliations:** 1 Pathology, All India Institute of Medical Sciences, Rishikesh, Rishikesh, IND; 2 Pediatrics, All India Institute of Medical Sciences, Jodhpur, Jodhpur, IND; 3 Pediatric Endocrinology, Super Speciality Paediatric Hospital & Post Graduate Teaching Institute, Noida, IND

**Keywords:** castleman disease, diagnostic pitfall, fine needle aspiration cytology, hyaline vascular type, lymphadenopathy

## Abstract

Castleman disease (CD) is an uncommon cause of lymphadenopathy. The role of fine-needle aspiration cytology (FNAC) as a diagnostic modality in this disease is not well established. Cytological features of CD have a considerable overlap with many reactive conditions. It has subtle morphological features; which if overlooked, may miss the diagnosis. A two-year-old girl presented with cervical lymphadenopathy. FNAC of the cervical lymph node showed features of granulomatous lymphadenitis. Excision biopsy revealed the hyaline vascular type of CD. Cytological smears were reviewed carefully and revealed indicators of CD. These included capillary fragments with adherent reactive lymphoid cells, plump endothelial cells and pale pink material admixed with germinal center cells. The collections of plump endothelial cells had been misinterpreted as granulomas previously. This report highlights the subtle cytomorphological pointers of CD. Careful scrutiny for these features could aid the cytologist in differentiating CD from other reactive and neoplastic disorders, thus avoiding cytodiagnostic pitfalls. This case study reiterates an important fact that for a lymph node lesion, histopathology plays a crucial role in differentiating mimickers and renders an accurate diagnosis.

## Introduction

Castleman disease (CD) is an uncommon cause of lymphadenopathy. The exact etiology of CD is unknown; however, pathogenic mechanisms like viral etiology and abnormal immune response have been postulated [1-6]. The role of fine-needle aspiration cytology (FNAC) as a diagnostic modality in CD is not well established; partly because its cytological features overlap with many reactive and neoplastic conditions, and partly due to lack of awareness of its cytomorphological features [1-3]. A definitive diagnosis of CD on cytology requires a high index of suspicion and awareness of the morphological features.

## Case presentation

A two-year-old girl presented with a gradually progressing swelling in the right side of the neck for one year. She gave no history of fever or cough. She had been started on anti-tubercular treatment (ATT) by a private practitioner. However, when the swelling failed to regress even after two months of ATT, she was referred to our Institute. On examination, there was a single, nodular swelling in the right anterior triangle of the neck measuring 5x5x4 cm. It was nontender, mobile, multinodular and felt rubbery in consistency. She had no swelling elsewhere.

**Figure 1:**
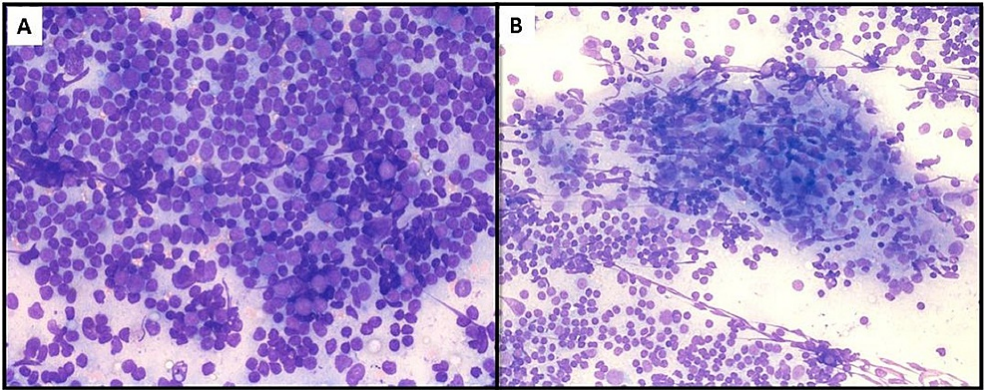
(A) Highly cellular smears showing a polymorphous population of reactive lymphoid cells - mature lymphocytes, centrocytes, centroblasts, immunoblasts and plasma cells (Leishman Giemsa stain, 400x). (B) Clusters of spindle-shaped endothelial cells mimicking epithelioid cell granulomas, admixed with lymphoid cells (Leishman Giemsa x 200).

The patient was referred for FNAC with a clinical suspicion of multi-drug-resistant tuberculosis. The FNAC was done using a 23-gauge needle and revealed cellular smears showing a polymorphous population of reactive lymphoid cells. There were numerous lymphocytes, centrocytes, centroblasts, immunoblasts and plasma cells (Figure 1A). Tingible body macrophages were scattered throughout the smears. There were aggregates of plump spindle cells forming ill-defined granuloma-like structures (Figure 1B). No necrosis, atypical lymphoid cells or acid-fast-bacilli were seen. Cytological impression was of granulomatous lymphadenitis; however, in view of the large size of the node and no response to ATT, complete excision and histopathological examination were advised.

**Figure 2:**
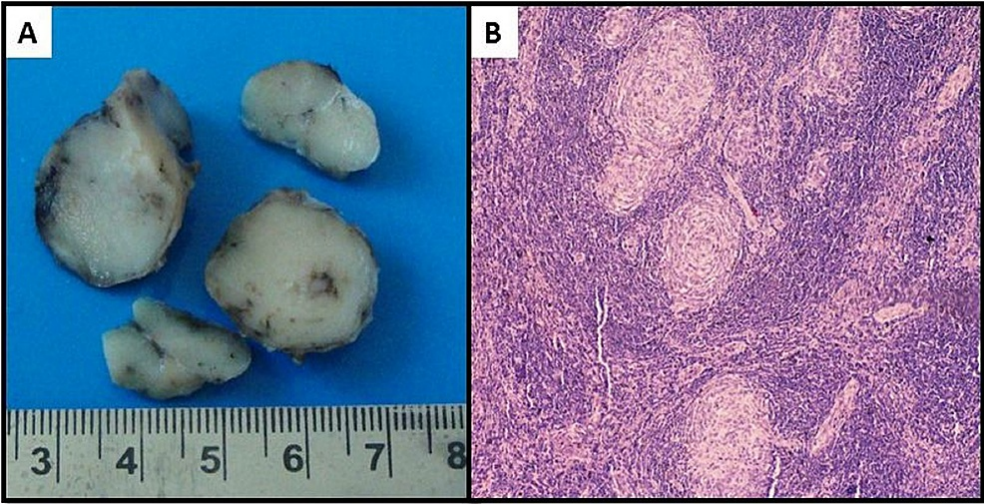
(A) Encapsulated multinodular mass measuring 4.5x2.5x2 cm. The cut section is gray tan, firm, solid and homogeneous. (B) Regressive, atrophic and hyalinized lymphoid follicles with concentric lymphocytic proliferation in an onionskin-like pattern. Few of the follicles are traversed by hyalinized blood vessel radially (hematoxylin & eosin stain x 100).

The excised node was encapsulated, multinodular and measured 4.5x2.5x2 cm. Cut section was gray-tan, firm, solid and homogeneous (Figure 2A). Sections revealed partial effacement of nodal architecture with numerous lymphoid follicles occupying the cortex and medulla, with the diminished sinusoidal region. Lymphoid follicles showed germinal center regression, prominence of hyalinized vessels within germinal centers and concentric lymphocytic proliferation in an onion-skin pattern. These follicles were traversed by blood vessels radially, giving a lollipop-like appearance (Figure 2B). Few follicles showed two or more hyalinized, atrophic germinal centers. The inter-follicular region showed increased vascularity and scattered plasma cells. Based on these features, a final diagnosis of hyaline vascular type of CD was made.

**Figure 3:**
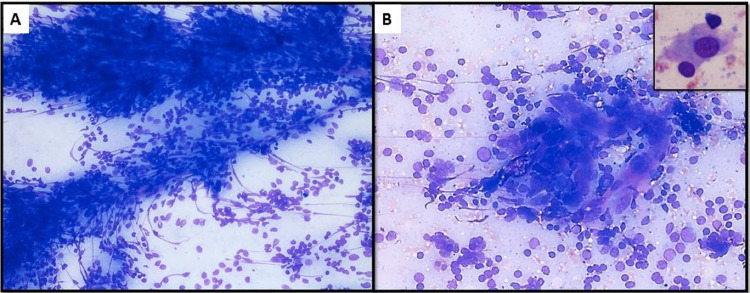
(A) Meshwork of capillaries with plump endothelial cells and adherent lymphoid cells (Leishman Giemsa x 200). (B) Hyalinized blood vessels appearing to penetrate an aggregate of germinal center cells, forming vague lollipop-like structure (Leishman Giemsa x 200). Inset shows a dendritic cell with abundant pale cytoplasm and eccentric nucleus (Giemsa stained smear x 400).

The cytological smears were reviewed and showed numerous collections of plump spindle-shaped endothelial cells, which had previously been misinterpreted as granulomas. The careful screening revealed subtle indicators of the specific diagnosis. These included numerous capillary meshes with adherent reactive lymphoid cells (Figure 3A) and occasional hyalinized blood vessels appearing to penetrate an aggregate of germinal center cells (a lollipop-like structure, Figure 3B). Giemsa stained smears showed pale hyaline-like material admixed with germinal center cells. There were few singly scattered follicular dendritic cells that were polygonal, with abundant pale cytoplasm, large, vesicular nucleus and inconspicuous eccentric nucleolus (Figure 3B, inset).

## Discussion

CD is a rare cause of lymphadenopathy which commonly involves the mediastinal and cervical nodes, and can be localized or multicentric [1,6]. There are two distinct histopathological variants of CD - hyaline vascular and plasma cell type. The hyaline vascular type is more common, presents as asymptomatic, localized lymph node enlargement; whereas the plasma cell type generally presents with systemic manifestations [6].

The histopathological features of CD are well known; however, literature pertaining to its cytological features is sparse. FNAC, which is otherwise a well-established diagnostic modality for lymphadenopathies, lacks a clearly defined role in CD [5]. Only a few reports in the literature highlight the cytological features of hyaline vascular type of CD [1-3,5]. Plasma cell type has non-specific cytological features and is therefore extremely difficult to diagnose on cytology.

The cytological features of hyaline vascular type of CD overlap with certain reactive and neoplastic conditions like reactive lymphoid hyperplasia (RLH), granulomatous lymphadenitis, Hodgkin lymphoma, Kimura disease, mantle cell lymphoma and thymoma [1-3]. The predominance of lymphoid cells; mature lymphocytes and germinal center cells can easily be mistaken for RLH. Spindle-shaped plump endothelial cells in clusters or scattered singly can resemble epithelioid cells and hence lead to an erroneous diagnosis of granulomatous lymphadenitis (which happened in our case). Even if the cytopathologist identifies the endothelial cells, their presence in a setting of the polymorphous lymphoid population with a predominance of eosinophils can cause confusion with Kimura’s disease. Plump endothelial cells in a background of RLH, especially in FNAC from a mediastinal mass in extranodal CD may strongly mimic a thymoma. Dendritic follicular cells can resemble mononuclear Reed Sternberg cells, especially when found in a background of RLH, which may lead to misinterpretation as Hodgkin lymphoma.

Thus, a spectrum of neoplastic and non-neoplastic disorders can mimic CD on cytology. However, there are subtle but definite morphological features that differentiate CD from its cytological mimics (Table 1). To reiterate, these include cellular smears with a predominance of lymphocytes, a generous admixture of germinal center cells, plasma cells and fragments of hyalinized capillaries closely intertwined with lymphoid cells. An interesting finding in the present case was capillaries entangled in follicular center cells, resembling the ‘lollipop-like’ structures (Figure 3B), which have been exclusively described in histopathology sections. Pale eosinophilic amorphous material within clusters of germinal center cells may also provide a diagnostic clue. A careful search for follicular dendritic cells, which stain positive for CD21 and CD35, also supports the diagnosis [5].

**Table 1:** Neoplastic and non-neoplastic mimics of hyaline vascular type Castleman disease on cytology CD - Castleman disease

Differential diagnosis	Reasons for diagnostic dilemma
Reactive lymphoid hyperplasia	Polymorphous lymphoid population, few cases in the literature, reported as reactive hyperplasia with increased capillaries
Granulomatous lymphadenitis	Scattered spindle-shaped endothelial cells or in clusters may mimic ill-formed granulomas
Hodgkin Lymphoma	Reactive lymphoid cells, plasma cells, few eosinophils. Dendritic follicular cells may be mistaken for Reed-Sternberg cells
Kimura disease	Reactive lymphoid hyperplasia, capillaries, eosinophils
Thymoma	Extranodal-mediastinal CD, hyalinized vessels, spindle cells

Through this case report, we have made an attempt to highlight those subtle, yet definite cytological pointers, the awareness of which can prevent a misdiagnosis on FNAC. This case study highlights the fact that pathological lesions can be missed or be diagnosed incorrectly on cytology due to various limitations of a cyto-diagnostic tool such as sampling error and overlapping cytomorphological features. Mimickers on cytology may mislead the pathologist resulting in an erroneous diagnosis resulting in incorrect clinical management.

The treatment of CD entails complete surgical resection of an involved group of lymph nodes. In the present case, cervical lymph nodes were excised. The patient was closely followed up and she did not have any recurrence till two years after excision.

## Conclusions

Diagnosing CD on cytology is difficult, but not impossible. A definitive diagnosis of CD on cytology requires a high index of suspicion and the cytopathologist should be aware of its subtle morphological indicators. Our experience with this case reiterates the fact that for a lymph nodal lesion, histopathological examination of an entire representative node is essential to identify the lesion and differentiate it from its mimickers.
